# Life Course, Green Space and Health: Incorporating Place into Life Course Epidemiology

**DOI:** 10.3390/ijerph13030331

**Published:** 2016-03-17

**Authors:** Jamie Pearce, Niamh Shortt, Esther Rind, Richard Mitchell

**Affiliations:** 1 Centre for Research on Environment, Society and Health, School of GeoSciences, University of Edinburgh, Edinburgh, Scotland EH8 9XP, UK; Niamh.Shortt@ed.ac.uk (N.S.); esther.rind@gmx.net (E.R.); 2 Centre for Research on Environment, Society and Health, Institute of Health and Wellbeing, University of Glasgow, Glasgow, Scotland G12 8RZ, UK; Richard.Mitchell@glasgow.ac.uk

**Keywords:** health, place, green space, environment, life course, health inequalities

## Abstract

Researchers interested in the relationships between place and health have been slow to incorporate a life course perspective, probably due to the lack of readily available historical environmental data. This hinders the identification of causal relationships. It also restricts our understanding as to whether there are accumulative effects over the life course and if there are critical periods in people’s lives when places are particularly pertinent. This study considers the feasibility of constructing longitudinal data on the availability of urban green space. The suitability of various historical and contemporary data sources is considered, including paper maps, aerial photographs and tabular land use data. Measures of urban green space are created for all neighbourhoods across the Edinburgh region of Scotland at various points during the past 100 years. We demonstrate that it is feasible to develop such measures, but there are complex issues involved in doing so. We also test the utility of the measures via an analysis of how accessibility to green space might alter over the life course of both people, and their residential neighbourhoods. The findings emphasise the potential for utilising historical data to significantly enhance understanding of the relationships between nature and health, and between health and place more generally. We encourage researchers to use data from other locations to consider including a longitudinal perspective to examine relationships between people’s health and their environment.

## 1. Introduction

There is growing international evidence that contact with, or access to, nature or green space may have beneficial implications for physiological and psychological health. Thematic and systematic reviews of observational and experimental studies provide tentative support for a connection between green space and a range of health outcomes including physical activity (and related outcomes), mental health and wellbeing, and perceived general health [1,2,3]. For instance, a recent systematic review of the relationship between objectively measured access to greenspace and obesity related outcomes (physical activity, weight status and health conditions related to elevated weight) concluded that a majority (68%) of papers demonstrated a positive or weak association between greenspace and obesity-related health indicators, although the findings were inconsistent across studies [4]. There is also evidence that socioeconomic inequalities in health may be narrower in places with better access to green spaces, compared with those with poorer access [5]. The pathways connecting green space and health are likely to include improving air quality, physical activity, social cohesion and stress reduction [3].

Although the evidence to date seems to indicate that contact with nature can promote health, a number of important questions remain, two of which are particularly pertinent to the current work. First, research evidence of a beneficial relationship between green space and health tends to rely on cross-sectional study designs. Such static study designs remain vulnerable to confounding, hinder the identification of causal relationships, and are subject to the limitations of reverse causation. Further, and related, if over time an individual moves between environments that are more or less supportive of good health, or there are significant changes in the neighbourhoods in which they live, then a single measure of residence, or other spatial location, is likely to misclassify environmental exposure and lead to an underestimation of the relationship between the environment and health.

A second important critique of green space and health work is the lack of work incorporating a life course perspective. Life course epidemiology has been instrumental in establishing that social, economic and cultural factors can build up over the life course (often referred to as accumulation models) to influence health later in life. Multiple exposures may cluster together in socially patterned ways and cumulatively damage or protect health; a consideration of the temporal ordering of, and inter-relationships between, the various exposures is important [6]. Alternatively there can be time-limited windows (critical periods) during the life course in which exposure can have adverse or protective effects on the development of health outcomes [7]. For instance, the well-known “foetal origins hypothesis” indicates that unfavourable social circumstances in early life and, in particular, poor maternal nutrition during gestation can lead to impaired foetal growth as well as heightened risk of hypertension, coronary heart disease and type 2 diabetes later in life [8].

Whilst life course epidemiology has revealed a great deal about how early exposure to factors such as socioeconomic conditions and childhood nutrition affect subsequent health outcomes, there has been less attention to incorporating “socio-environmental” perspectives, and in particular the role of factors that are rooted in place. Life course approaches that incorporate longitudinal information relating to the places in which people have lived their lives could not only provide insights into the accumulation of risk over time but might also enhance our understanding of the critical periods of people’s lives whereby place-based constructs are particularly pertinent. The few environment and health studies to have incorporated a life course perspective have been restricted to examining exposure to area-level social economic circumstances, usually adopting proxy measures from the corresponding national census [9,10,11,12,13]. International evidence suggests that health outcomes at older ages are related to the nature and quality of local environmental elements, including public transport, the public realm, community resources (e.g., shops, banks, post offices and cafes), green spaces and trees, social capital, and community networks [14], yet these issues are rarely considered using longitudinal data.

In sum, both of these critiques (failure to establish causality and inadequate attention to life course perspectives) point to the need for long-term longitudinal investigations of green space health relationships. To date this has rarely been achieved, likely because of the significant challenges of assembling historical environmental data. A longitudinal study design may assist in inferring causality and revealing the relative influence of both health and social selection. This type of approach may also facilitate an exploration of the accumulative influence of contact with (or exposure to) green space across the life course as well as the establishing whether certain populations are sensitive to green space during particular “critical windows” in life.

### Aims and Rationale

The aim of this methodological paper is to examine the feasibility of incorporating measures of green space into a life course epidemiology framework. A prerequisite for this approach is longitudinal information relating to the various places in which a cohort of individuals has resided over the course of their lives. Using the Edinburgh region of Scotland as an example, we provide a heuristic for developing neighbourhood green space measures at multiple time points over the past 100 years. In doing so, we examine the suitability of various historical and contemporary data sources including censuses, historical maps, aerial photography and historical land use data.

The strategy for collecting green space data has been purposively designed to enable future integration with the Lothian Birth Cohorts (LBC) of 1921 and 1936 [15]. This data set provides detailed information on a cohort of people born in the Lothian area of Scotland in the first part of the twentieth century. It collates health and socio-demographic information gathered at various points through their lives. In this paper we examine the feasibility of supplementing the current data with information relating to the respondents’ residential environments at multiple time points. The cohorts are a revitalisation of the Scottish Mental Surveys of 1932 and 1947 which tested the “general intelligence” of every child born in 1921 and 1936 attending school in Scotland in 1932 and 1947. LBC 1921 members were then resurveyed at ages 79, 83, 87 and 90, and LBC 1936 members were resurveyed at age 70, 73 and 76. The collected outcomes include a range of physical and psycho-social aspects of wellbeing in old age alongside demographic, psycho-social and lifestyle factors. Cohort members are geographically dispersed across the Lothian region, ensuring an assortment of environmental types. Environmental variables will be constructed for all neighbourhoods across the city at various points during the respondents’ lifetimes. We consider the utility of this approach and present some opportunities for new research that could be addressed using these ideas. This strategy will enable us in the future to establish (a) the cumulative effects of exposure to green space (b) whether green space exposure at critical windows earlier in life has subsequent health effects, and (c) whether negative green space exposures in early life affect social trajectories in later life, in turn influencing health and wellbeing. Whilst the focus for the future empirical component of the study is the Lothian region of Scotland, the work has broader conceptual and methodological implications and the consideration of these concerns forms the basis for this paper.

## 2. Materials and Methods

We aimed to identify all data pertaining to publically accessible green spaces spanning the time period covered by the Lothian Birth Cohort (from the early twentieth century to the present day). Our search strategy was two pronged. First, we identified appropriate archives through meta-search tools, including JISC content (digital collections and archives for learning, teaching and research) (http://www.jisc-content.ac.uk/), the National Archive ARCHON Directory (http://webarchive.nationalarchives.gov.uk/20080108014935/nationalarchives.gov.uk/archon/) and the Scottish Archive Network Catalogue (http://www.scan.org.uk/aboutus/indexonline.htm). From these tools we identified relevant collections, filtered by location (City of Edinburgh, Lothian area), subject area (e.g., social sciences, geography and environment, architecture and planning) and record type (e.g., maps, local authority reports, aerial photography).

Second, we inspected each collection in turn (either remotely in the case of web-based collections or in person), identified relevant material and assessed the suitability of the material. The multitude of potentially relevant data sources was vast and included historical maps, aerial photography, and civic survey plans. In order to guide our search we used a scoping procedure, based on several criteria selected to enable longitudinal analyses of the relationships between green space and health using the LBC data (Table 1). Material that failed to meet one or more categories was excluded. After identifying relevant public green space data, the subsequent challenge comprised the integration of these data into a Geographical Information System (GIS). Using GIS techniques we scanned, geo-referenced and digitised identified datasets which facilitated the extraction of relevant information. Doing so allowed us to create a comparable public park measure from 1900 to the present day.

### Simulating the Effect of Green Space Change Using a Pseudo Cohort

For this paper, we illustrate the utility of these data by using them to simulate how a cohort of residents might experience change in access to open spaces over time. Due to confidentiality constraints it was not feasible to use the geographically-specific data in this mapping exercise as it would reveal the location of the LBC respondents. Our future longitudinal work assessing the health implications of green space exposure will match precise residential address of each cohort member with the corresponding local green space measures. As a demonstration of the methods we created a simulated cohort for the Edinburgh area, based on the Lothian Birth Cohorts 1921 (*n* = 550) and 1936 (*n* = 1091) [15] using the postcode districts in which they lived (geographically smaller areas will be available for subsequent analyses). For the 1921 (*n* = 430) and 1936 (*n* = 875) cohorts we used contemporaneous Ordnance Survey maps and Post Office plans [16] to randomly place the correct number of cohort members within each postcode district onto the contemporary street and housing network. This approach maintains the confidentiality of the cohort members whilst at the same time helps to demonstrate the utility of our approach. To enable comparisons across time, we excluded cohort members residing beyond the extent of the open space surveys (*i.e.*, the Edinburgh region). In order to simulate how neighbourhood green space availability changed over time (rather than how people’s access to green space altered over the life course) we assumed residents had remained at the same address throughout their lives. Although this approach is likely to underestimate variations in life time green space “exposure” it provides an indication of how urban green space has evolved. Future work will integrate green space data with information on the residential history of each respondent to examine green space accessibility changes over the respondents’ lives, and the implications for various health-related outcomes.

We created two buffers around each park. The buffers sizes were based on recent guidelines proposed by the Edinburgh Open Space Strategy [17] which indicates that residences should be within 400 metres walking distance of a green space of at least 500 square metres, and within 800 metres walking distance of a green space of at least two hectares. For each of the survey years we then identified whether the cohort members resided within the 400 metre and 800 metre buffer zones at each time period.

## 3. Results

Our evaluation of a range of potential data sources demonstrated that it was feasible to develop longitudinal small area measures of green space that have potential utility in life course epidemiology. Data on public parks in Edinburgh were available from a variety of sources, including development reports and civic surveys, historical (paper) maps (e.g., Ordnance Survey), and digital GIS-compatible records (e.g., shape files) and covered time points between 1914 and 2009. Contemporary data were readily available in digital format but data for other time points required significant processing to transform to digital format (Table 2).

We identified four open space surveys that included details of public parks in Edinburgh in 1914, 1949, 1969 and 2009. The surveys were more appropriate for our purpose than the standard land use maps produced by the national mapping agency (Ordnance Survey) as they provided a more detailed description of the different types of green space within the urban area. The 1914 M’Hattie Open Space Survey included major public parks, playgrounds, bowling greens, cemeteries, jail enclosures as well as small road enclosures [18]. The 1949 Abercrombie Survey [19] provided information on a variety of open spaces, including public recreation grounds, as well as private or institutional open spaces. The 1969 Town Planning Department’s open space survey [20,22] included a detailed description of the distribution of public and private open spaces and recreational facilities, as well as a survey including information on the popularity of Edinburgh’s parks. It also included information on the perceived quality of each park in the study area (e.g., maintenance, infrastructure and litter), but the lack of comparable data through time restricted the utility of these data. In comparison to the earlier surveys, the 2009 open space audit is the most comprehensive, both in terms of spatial extent and in terms of detail about different types of open spaces. It also included a quality assessment [21,23]. We scanned and geo-referenced the 1949 and 1969 surveys in order to integrate the data within a GIS. At the time of our data collection process, the 1914 open space survey was only available as reference material, and due to the delicate condition of the plan it was neither possible to photocopy nor to take any photographs of the map. We therefore manually recorded the location of different parks, recreation grounds and open spaces, their approximate shape and acreage. These details were available in a separate table within the original report. Using GIS techniques this information was then transformed into a digital format.

**Figure 1:**
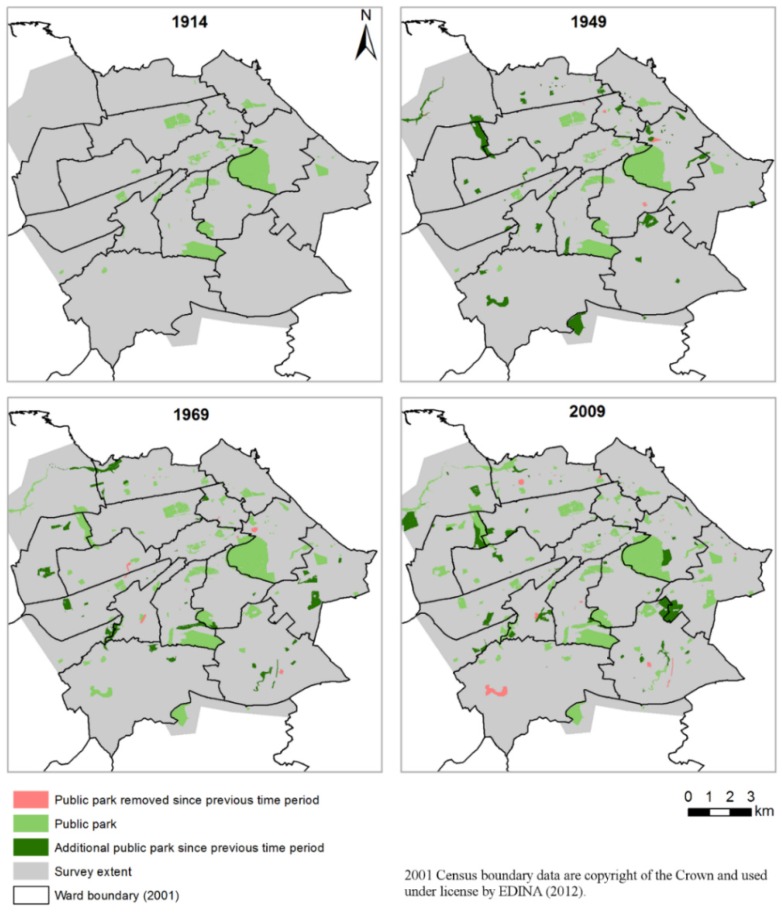
Mapping the public parks in Edinburgh in 1914, 1949, 1969 and 2009.

Each of the four data sources recorded the various types of open spaces slightly differently. For each of the four years, we included areas of open space where the label suggested the areas were publically accessible including “public parks”, “public open space” and “public recreation grounds”, and in later years smaller spaces such as “playing fields”, or “residential playgrounds or amenities”. The 1969 and 2009 surveys identified whether each open space was accessible to the public but the earlier surveys did not. For the earlier years we assumed all open spaces labelled as a public park (or similar) were accessible, and other spaces that were identified as accessible in 1969 and 2009 were also accessible in 1914 and 1949. The 2009 data were available in GIS format and we used this most recent data set as our base map. For the earlier years, we amended the data set (added or removed polygons) according to the information identified in the 1914, 1949 and 1969 surveys. By combining and integrating each of the data sources we created consistent measures of Edinburgh’s public parks between 1914 and 2009 (Figure 1). The maps shows the growth in the provision of public parks in the Edinburgh areas largely reflecting the expansion of the urban area from the 1930s onwards. By 1969 most of the post-war slum clearances had been completed and new areas of social housing constructed; these changes are reflected in the increase in green space between 1949 and 1969. Whilst the maps generally demonstrate an increase in the provision of public parks, there are examples of locations where public parks are no longer present (see for example a public park located in the south west corner of the city in 1949 and 1969 but no longer present in 2009).

**Figure 2:**
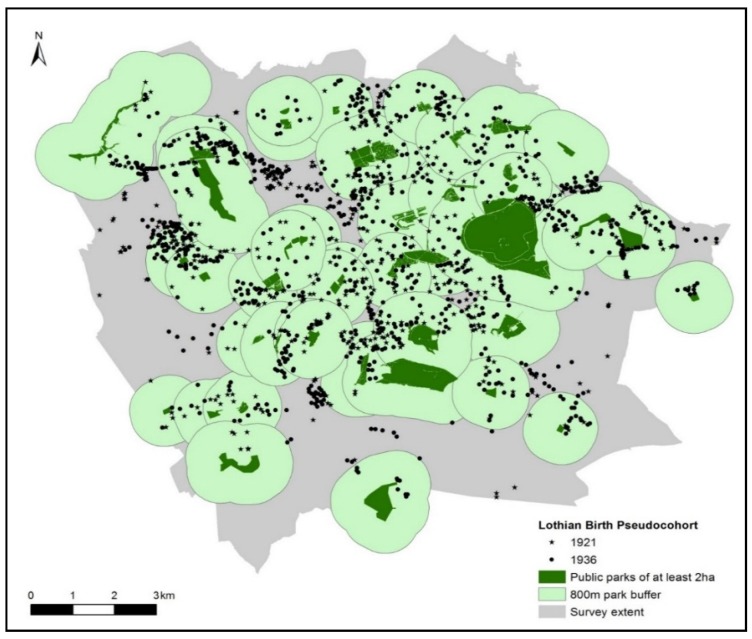
Distribution of the pseudo cohort in relation to public parks in the Edinburgh region in 1949.

To demonstrate the utility of these data, we now illustrate how the availability of green space might have changed over time for members of the Lothian Birth Cohorts. Figure 2 shows an example of the distribution of the pseudo cohort in relation to the 800 metres buffers for parks of at least two hectares in 1949. Table 3 summarises the percentage of the individuals in the pseudo cohort living within the recommended distances to public parks at each point. The results suggest that, from 1946 onwards, a greater proportion of cohort residents were within the recommended maximum distance to a green space. For example, between 1949 and 2009 the proportion of the pseudo cohort population residing within 400 m to a park of at least 500 square metres rose from 58.5 to 74.8 percent. Using the same park access criteria, our simulation suggested that 22 percent of the cohort members had no access to a park over the whole of the study period, whereas 33 percent of members always had access (Table 4). In summary, the results based on the pseudo cohort exemplify firstly how data in the Lothian Birth Cohorts could be considered in relation to the development of public parks over time and secondly the increased availability of parks in Edinburgh over the past century.

## 4. Discussion

A significant body of research has considered the ways in which environments influence and mediate a multitude of health and related outcomes. Yet few studies considering place-health relations have used a longitudinal design. Hence, whether and how residence in different kinds of places across the life course affects health remains poorly understood. Similarly, few life course researchers have considered the multiple ways in which place affects health and wellbeing at various points during life. As Diez Roux and Mair note, strategies that will enable linkage between meaningful historical area-level information and cohort data are an important research need [24]. One of the few studies to adopt a longitudinal design identified variations in the association between green space availability and mental health outcomes across the life course in England [25]. The authors found that benefits for men of greater availability of local green space were apparent primarily in early/mid-adulthood, but benefits for women occurred later in life, and varied by density of green space. The authors highlight weaknesses in current understanding of *which* green spaces matter at different stages in life and *why*, describing this area as *“severely under-researched”*. This omission is likely due to the difficulty in identifying and obtaining geographically-detailed historical data, and inconsistencies in reporting units over time [26].

Our study has considered the feasibility of incorporating measures of green space into life course epidemiology, and examined the associated conceptual and technical challenges of gathering data about places to do so. Focusing on public green space, our findings suggest that whilst identifying and operationalizing environmental data over time is challenging and often labour intensive, it is feasible to develop longitudinal small area measures which can be potentially linked with cohort information. With the regular development of new cohort data sets that are often collected in a single urban jurisdiction, there is potential to supplement these data with longitudinal environmental data including green space. At the same time there is growing interest amongst social historians, demographers and others in developing mapping resources for tracking and understanding social and economic changes across urban areas including health-related aspects of the urban infrastructure such as housing, transport and community resources see for example the Historical Spatial Data Infrastructure of the City of Madrid (HISDI-MAD); [27]. Hence, greater collaborative endeavour between public health researchers, social historians, and those working on mapping social changes is likely to be productive in developing our understanding of the relations between place and health [28]. We provided examples of data sources which may be used to develop a spatial history of public park development in Edinburgh between 1914 and 2009.

The novel data sets we collected have utility in enhancing understanding of the green space-health relations specifically, and the role of place in understanding health more generally. By combining our measures of green space at various times points over the past 100 years with information (including residential history) on a cohort of individuals residing in the Edinburgh region over this period a number of research possibilities arise. First, and in particular, it will be feasible to examine the influence of green space at different points in the life course on subsequent health outcomes. For instance, we can consider how green space exposure in adolescence influences a range of learning-related outcomes and identify the significance of these effects for health later in life. It is plausible that green space exposure in formative years can affect physical and mental health outcomes later in life [11,29]. It will also allow us to examine issues such as shifting life circumstances, e.g., educational and employment opportunities, as well as personal orientations to green space and nature influence engagement [30]. For instance, childhood participation in nature-based activities may represent the start of “nature acculturation” [31], with different types of nature interactions shaping those sought out as adults [32,33,34]. This acculturation process may continue, with changes in adulthood (e.g., parenthood, relocation, income shifts, and reduced mobility) also influencing opportunities for, and interest in, green space interaction. We have limited understanding of such dynamics, in turn, limiting the effectiveness of public health improvement activities. In particular, we know little about the role of individual characteristics and circumstances in seeking out and experiencing green space as a wellbeing resource, how this might influence inequalities in health and wellbeing, and in particular how this varies through the life course [30,35,36]. The integration of green space measures at various time points and LBC data collected during childhood and later in life will enable us to better understand the (dis)enabling factors (personal and environmental) affecting respondents gaining benefits from green space provision.

Second, for the first time it will be possible to consider the cumulative influence of green space throughout the life course. This work can include examining the influence of cohort members moving between environments with varying degrees of green space; importantly this is work that can strengthen causal evidence. The migration and health literature, especially work concerned with health-related selective mobility, supports the notion that residential movement can result in substantial alterations to an individual’s social and physical milieu [37,38]. Third, it will be feasible to identify whether there are time-limited critical periods during the life course in which green space can affect the development of health outcomes. Life course approaches which incorporate longitudinal information relating to the places in which people have lived their lives could provide insights into the accumulation of risk over time but might also enhance our understanding of the critical periods of people’s lives whereby place-based constructs are particularly pertinent. These assertions have rarely been tested and the lack of work in this area remains a significant impediment to understanding health-environment relations. These and other possibilities demonstrate the analytical opportunities of combining historical data on green space (and potentially other environmental characteristics) with longitudinal data that includes information on health outcomes.

Our study had limitations. First, we found that there are some time periods for which historical green space data for Edinburgh were not readily available (e.g., the 1930s). The challenges of obtaining historical data to examine changes in urban neighbourhoods, including problems with what is represented, the spatial and attribute accuracy of data, and the purpose for which the data were collected in the first place, have been noted by those working with GIS in historical contexts [39]. We also found that, for the earlier time points, obtaining and processing the data was time consuming and, at times, technically challenging. Some data sets were difficult to source due to copyright restrictions, bureaucracy in gaining access, or the fragility of the original paper-based resource. Second, there were also technical challenges associated with scanning, geo-referencing and digitising the data for incorporating into a GIS, as well as ensuring that the measures were comparable over time. Our investigation demonstrated that whilst there were relatively good historical records for the Edinburgh urban area, data for the rural and semi-rural areas of the wide Lothian region were less readily available. Third, our focus was the Edinburgh region of the UK and how generalizable our findings would be to other areas within the UK or in other countries is unclear. However, many of the data sources we identified are available for other parts of the UK and other cities also have a range of archival material that is likely to be comparable to what is available in Edinburgh. The information we have relates to places of residence and we do not have information on all of the key locations that might be important in understanding their subsequent health (e.g., workplace). Finally, it is feasible that types of green space other than public parks may also influence health including ambient green space, private parks, allotments and gardens. It is potentially feasible to collect information on a wider set of green spaces types; this is a possible area for future research.

## 5. Conclusions

In conclusion, the incorporation of geographically-specific longitudinal information on the health-related characteristics of areas offers considerable analytical potential for researchers with an interest in the links between place and health. Our work demonstrates that whilst this ambition is technically challenging, it is feasible. The next stage of our research will involve collecting a wide set of longitudinal geographically-specific data including information on voting patterns, retailing, crime and social context. This geographical information will be appended to data on the cohort members to enable us to investigate the role of place at various points in the life course in explaining health in later life.

Local environments, and hence local risk factors, can change rapidly, and this is likely to have profound implications for health, wellbeing and inequalities; further research is needed to understand these changes. We encourage health researchers concerned with geographical or environmental accounts to adopt a life course of place approach and develop small area longitudinal measures. With a wider set of environmental measures it will be possible to consider whether health-place relations are formed through steady accumulative “exposure” over time (e.g., gradual improvements to local resources and infrastructure) and/or due to critical moments with sudden and perhaps profound implications for health (e.g., closure of an industry dominating local employment opportunities).

## Figures and Tables

**Table 1:** Criteria for data selection.

**Time**—the time frame covered the period from the early 20th century onwards. Data were required either in form of a time-series, or multiple data sources with potential to develop consistent measures through time
**Spatial Extent**—the datasets covered the Edinburgh urban area and/or the Lothian region
**Digitisation**—data sources could be transformed into a digital format for integration into a Geographical Information System (GIS)
**Scale & Quality**—the maps, aerial photography and other material were at a scale, and of a quality, which enabled the identification of the location and geographical extent of local parks

**Table 2:** Available data sources for capturing local green space in Edinburgh over time.

Identified Measures	Years	Data Type	Spatial Unit/Scale	Digitally Available	Source
Acreage and location of parks, recreation grounds and open spaces in Edinburgh	1914	Paper map	1 mile = 2 inches	×	Open Space Survey [18]
Acreage and location of public open spaces and public recreation grounds in Edinburgh	1949	Paper map	1 mile = 2 inches	×	Edinburgh Civic Survey [19]
Acreage and location of public parks, golf courses and open spaces in Edinburgh	1969	Paper map	1 mile = 1.2 inches	×	Open Space Survey, City of Edinburgh [20]
Acreage and location of public parks & gardens, and accessible open space in Edinburgh	2009	GIS file	zoomable to <1:500	√	Open Space Audit 2009 [21]

**Table 3:** Availability of public parks amongst the pseudo cohort for the four survey years.

Availability of Public Parks	Pseudo Cohort (*n* = 1305)							
	*n*	%	*n*	%	*n*	%	*n*	%
	in 1914		in 1949		in 1969		in 2009	
of at least 500 m^2^ within 400 m	473	36.2	763	58.5	908	69.6	976	74.8
of at least 2000 m^2^ within 800 m	660	50.6	1072	82.1	1176	90.1	1228	94.1

**Table 4:** Public park availability amongst individuals in the pseudo cohort between 1914 and 2009.

Public Park Availability	of at Least 500 m^2^ within 400 m		of at Least 2000 m^2^ within 800 m	
	*n*	%	*n*	%
Availability throughout	436	33.4	653	50.0
Only had availability in 1949 & 1969 & 2009	298	22.8	415	31.8
Only had availability in 1969 & 2009	142	10.9	104	8.0
Only had availability in 2009	91	7.0	56	4.3
No availability throughout	291	22.3	66	5.1
Other	47	3.6	11	0.8
Total	1305	100.0	1305	100.0
